# Supporting the Development of Science Pre-service Teachers’ Creativity and Critical Thinking in Secondary Science Initial Teacher Education

**DOI:** 10.1007/s11165-023-10104-x

**Published:** 2023-03-03

**Authors:** Karen Marangio, Jared Carpendale, Rebecca Cooper, Jennifer Mansfield

**Affiliations:** 1 School of Curriculum, Teaching and Inclusive Education, Faculty of Education, Monash University, 19 Ancora Imparo Way, Clayton, 3800 Australia; 2 Institute of Education, Massey University, Palmerston North, New Zealand

**Keywords:** Creativity, Critical thinking, Science education, Pre-service teachers, Teacher educators

## Abstract

Creative and critical thinking (C&CT) capabilities are essential qualities of future ready scientific literate citizens. As teacher educators, developing C&CT in science pre-service teachers (PSTs) requires supporting PSTs’ development of C&CT, in addition to supporting their understanding and capacity to teach for development of C&CT in their future school science students. In this study, four secondary science educators critically reflected on the development of our professional knowledge and practice for supporting secondary science PSTs’ understanding of, and capacity to teach, C&CT as future teachers of science. Meeting transcripts, reflective journaling and curriculum documents were inductively analysed for key themes, utilising an iterative approach with multiple cycles of review. Findings showed that integrating C&CT in explicit ways in our teaching and assessment tasks was not as straight-forward as initially imagined. Three themes were identified, showing how our thinking evolved, namely (1) becoming sensitised to C&CT in our science ITE practice; (2) developing a shared language and understanding for science education; and (3) illuminating the conditions for teaching C&CT. A recurring feature in all themes was the value of tensions for sensitising us to specific aspects of C&CT and its teaching. We offer recommendations for others seeking to develop science PSTs’ C&CT.

## Introduction

In acknowledgement of the significance of creativity and critical thinking (C&CT) to cope with an uncertain future, the Organisation for Economic Co-operation and Development (OECD) conducted a large-scale international study titled “Fostering and Assessing Creativity and Critical Thinking in Education”. This OECD study aimed to support higher education institutions to enhance the quality of teaching to foster and assess students’ C&CT. The initial teacher education (ITE) secondary science education team (teacher educators (TEs) and authors of this paper), along with teams from other faculties at Monash University, participated in this interdisciplinary project. The directive was to revise curriculum and assessment tasks to explicitly promote and enhance C&CT development. In response, we met regularly to reflect on the development of our practice for facilitating secondary science pre-service teachers’ (PSTs’) C&CT for their future teaching of science. This work aligned well with science education as C&CT are integral aspects of scientific endeavour. Berry (2021) argues that the relative absence of organised professional learning can provide TEs with opportunities to take ownership of their professional learning in rich and contextualised ways. Since this OECD work aligned well with science education, we took this opportunity to learn how to draw from and restructure our existing knowledge in ways to enable our teaching in ITE science education. During our regular meetings, we realised that this experience was challenging our own thinking in significant and meaningful ways. Through these realisations, we explored our own professional learning for teaching science PSTs, which was guided by the following research question:

In what ways was our professional knowledge challenged, stimulated, or developed, as we supported secondary science PSTs to develop their own C&CT capabilities as future teachers of science?

## Literature Review

### Understanding Creativity and Critical Thinking

C&CT are commonly identified as key capabilities for future ready students, yet developing these skills is seldom attended to effectively in school and university settings (Marquis et al., 2017; Vincent-Lancrin et al., 2019), especially when there is a lack of consensus about what constitutes C&CT (Kind & Kind, 2007). Teaching for C&CT requires educators to understand the nature of these attributes and the capacity to recognise and frame them in practice. Complexity exists for TEs as it is not enough to simply develop PSTs’ C&CT skills. Rather, TEs must also support their students to develop their capacity to teach about, and assess, C&CT to their future students (Lorencová et al., 2019). This is particularly relevant for science teachers given the integral link between C&CT and scientific endeavour (Mansfield & Gunstone, 2021); thus, it is essential to include C&CT in primary and secondary science education when espousing science as a human endeavour. To explore this complexity, it is useful to first explore conceptions of C&CT and their role in scientific endeavour.

There are different approaches to conceptualising creative thinking. Creativity is a term often used interchangeably with the concepts of “originality, innovation, divergent thinking and idea generation” (Ellerton & Kelly, 2021, p. 11); however, each of these terms defines different skills and attributes. Creativity can be seen to involve the movement from thought (i.e. imagination and ideation), to actions, to novel and adaptive outcomes or displays, as a process that is transformative, and necessarily involves risk (Ellerton & Kelly, 2021). Dennett (2013) suggests creativity also includes appropriateness, where novelty is not just about doing something new. It is about usefulness and adaptiveness within a specific context. Craft and Hall (2014) suggest creative activities involve practical, social, intellectual and values-based practices and approaches. Scientific creativity is evident in scientific endeavour as science involves recognising the need for new knowledge to solve contemporary challenges in society (e.g. Hadzigeorgiou, 2016; Kind & Kind, 2007). This requires creativity to formulate research questions and develop novel investigations to generate new data, which can be used as evidence to generate new theories. Creativity is also necessary to make new connections between disciplines, a willingness to take risks and play with new and unusual data and insights and to communicate findings to a variety of audiences.

In contrast, critical thinking permeates aspects of creative thinking and action, as “*critical thinking* includes the effective synthesis and analysis of all gathered and generated information and alternatives to inform decisions leading to problem resolution throughout the creative process” (Ellerton & Kelly, 2021, p. 17, emphasis in original). Ellerton and Kelly (2021) characterise critical thinking by defining skills, values and virtues embodied by critical thought. Skills include argument constructions and evaluation, along with being able to collaborate, reason and communicate. Values include the ability of an individual to apply aspects of inquiry, such as accuracy, precision, simplicity, reproducibility, coherence and relevance, rather than focusing on the process of inquiry itself. Many of these values are particularly relevant in science for the generation, interrogation and use of data as evidence. Inquiry virtues, such as being open minded, tolerating others’ views, intellectual honesty, humility and charity, are also necessary in science to extend one’s ability to seek new possibilities, reinterpret old problems and question personal assumptions. Considering critical thought in this way acknowledges that critical thinking, especially in science, involves more than simply understanding the nature of problem solving in a particular domain or the use of higher order thinking skills.

### Creative and Critical Thinking in Higher Education

Teaching for the development of C&CT is seen as a global imperative to nurture independent, critically discerning and adaptable global citizens (Vincent-Lancrin et al., 2019). Yet, the teaching of C&CT has been criticised as limited and ineffective in higher education due to staff and student attitudes, along with contextual realities, such as overemphasis on impact and performance, which discourages risk-taking (Marquis et al., 2017). Other barriers include ambiguity around definitions of C&CT (Egan et al., 2017) and science-specific thinking and bias, such as scientists seeing creativity at odds with “the scientific method” (Walsh et al., 2013), or creativity being seen as “the special purview of the arts” (Marquis et al., 2017, p. 223), leading to an “arts bias” view of creativity (Patston et al., 2018). The issue of “arts bias” and creativity is particularly relevant when considering the nature and role of creativity in science education, as developing scientific literacy requires understanding and awareness of science as a creative human endeavour which requires both C&CT (e.g. Sammel, 2014).

Research related to C&CT during ITE courses have focused on the use of creativity to innovate teaching, such as creative pedagogies that involve risk taking; yet higher education settings are risk averse, which restricts creativity (Patston et al., 2021). Increasing accountability through assessments, ranking systems, “fear of failure” (Mansfield & Gunstone, 2021) and push back from students may influence those in higher education to opt for more traditional, less creative pedagogies which fail to model or privilege creativity, leading to risk aversion (Watson, 2018). For critical thinking in ITE, economic pressures are shifting the focus away from “critical thinking” skills to the more generic “employability skills” (Davies & Barnett, 2015). Research into the development of critical thinking skills in university graduates suggest development is not automatic (Janssen et al., 2019), critical thinking skills are rarely taught explicitly and the development of critical thinking in students relies on the quality of instruction (Janssen et al., 2019). ITE for science PSTs needs to provide opportunities for them to develop awareness of their beliefs and values about C&CT as well as develop C&CT in their future secondary school students of science, along with emphasising how C&CT are integral to science.

### Developing Science Pre-service Teachers’ Creative and Critical Thinking

In ITE, the development of critical thinking skills in students has been examined; however, less attention has been paid to the development of creativity, and TEs’ knowledge about teaching C&CT to PSTs (Lorencová et al., 2019). For creativity, Jeffrey and Craft (2004) distinguish between *teaching creatively* and *teaching for creativity* (emphasis in original), where teaching creatively relates to using imaginative approaches to engage learners whereas teaching for creativity is defined as pedagogical approaches which encourage students to develop their own creative thinking and behaviours. The development of C&CT is more likely to occur when “the teaching of these skills is explicit (where learning goals and explanations of knowledge and skills are clearly outlined for the students), rather than implicit (where such explanations are not made overtly)” (Lorencová et al., 2019, p. 846). To teach C&CT explicitly, TE’s must first see value in teaching C&CT, then cultivate their own understanding of the skills (Lorencová et al., 2019). To support PSTs’ C&CT development and awareness, Lorencová et al., (2019) found support for active and collaborative learner centric strategies, such as self-learning, discussion, authentic situations and mentoring/feedback. When teaching, Lorencová et al., (2019) suggests the need to cater for “personal (i.e., students’ preferences), methodological (i.e., tools, duration, feedback), and contextual (i.e., classroom climate, supportive initiatives) features” (p. 854) through complex teaching procedures, creative preparation, and the use of modern technologies. Students must also feel that the class environment allows them to be unconventional and take risks (Skinner et al., 1994). This shift from traditional modes can be challenging for TEs and PSTs, as it requires a shift in mindset about who is responsible for who’s learning, especially within complex and time poor ITE landscapes, along with changes to classroom contexts to allow for risk taking. Assessment of C&CT in higher education has been through non-standardised means, such as various forms of feedback, reflection and self-assessment (Lorencová et al., 2019). Such means raise questions about how student work might be assessed. These are some of the challenges that we faced with our involvement in the OECD project, which led us to explore changes to our own thinking and practice.

## Methodology

This study was positioned within a small-scale research paradigm (Knight, 2002). While this positioning can be seen as a limitation, this methodological approach allowed for different ways of thinking about an experience to emerge (Poulson & Wallace, 2003). Focusing on a small group of participants for this study allowed for in-depth exploration of participant voices as sources of information (Guest et al., 2006). Given the nature of the research question, a qualitative research approach with an interpretive lens was appropriate, particularly as participants’ lived experiences were sought to analyse for themes (Elliott and Timulak, 2005).

### Participants and Context

Participants in this reflective study were four science TEs teaching across five ITE secondary science education subjects in Victoria, Australia^1^ (Biology, Chemistry, General Science, Physics, and Psychology). Each subject spanned one semester and consisted of eight subject-specific workshops and eight common weekly seminars, with one common assessment task (the first assessment task was the same for all secondary science PSTs, but contextualised for the specific science subject). The assessment task and seminars were key aspects where changes were made in response to the OECD project, including using The Domain General C&CT rubric.^2^Changes included re-writing the assessment task to emphasise aspects of C&CT, re-structuring the marking rubric, and revising the common seminars to explicate aspects of C&CT for teaching science.

### Data Collection

Qualitative data were collected over two years, consisting of the following:

• Meeting (*N* = 12) recordings, which focused on planning and reflecting on ways to support secondary science PSTs’ development of C&CT
• Reflections submitted to the OECD (Dec 2019; May 2020)
• Individual reflective teaching journals
• Curriculum documents, including unit schedules, assessment tasks, and marking rubrics

Data sets were analysed at the beginning of each year, enabling holistic thinking about changes in our C&CT understanding and teaching practices for science education as our understanding developed over time. Note, data collected for this study took place during the COVID-19 pandemic. Consequently, we had to make a rapid change from teaching on campus to teaching online (see Cooper et al., 2020; Carpendale et al., 2020; Marangio et al., 2021) and we remained online throughout the two years.

### Data Analysis

Data were analysed using an inductive thematic approach (Bryman, 2016) to understand changes in professional knowledge and practice. Analysis was iterative, with multiple cycles of review over a sustained period, leading to continual refinement of the themes. The first analytical cycle was undertaken individually after the first year of teaching. Initial results were subjected to collaborative discussion to consider each other’s interpretations of the data. This process led to further categorisation and refinement of themes. The analysis process was repeated after the second teaching semester. Analysing the data in this way identified three salient themes, which are now explored.

## Findings

Findings are presented around the three themes: (1) becoming sensitised to C&CT in our science ITE practice; (2) developing a shared language and understanding for science education; and (3) illuminating the conditions for teaching C&CT to future science teachers. These themes illustrate how our professional knowledge as science TEs was challenged, stimulated and developed as we worked together to explicitly embed C&CT in our teaching and assessment to support our PSTs to develop their own C&CT as future teachers of secondary school science.

### Theme One: Becoming Sensitised to Creative and Critical Thinking in Our Science ITE Practice

Initially, we noticed whether (or not) we were explicitly teaching C&CT, and then, as we became more deliberate in our practice, we were challenged by what it means to explicate C&CT in science education. Before the start of the 2020 semester, we reconsidered our secondary science common assessment task in light of assessing C&CT, guided by The Domain General C&CT rubric from the OECD project. We intended to create a new assessment task for the OECD project. However, as we interrogated the purpose of our original assessment task, we identified that aspects of C&CT were already inherent in the task, but we were not explicitly assessing creativity.

Creativity was very important, but interestingly, we had not been explicit in the assessment… By not including creativity we were placing a lower value on it. This realisation was an eye-opening experience for us*.* (Karen, Reflection, Dec 2019).

Thus, we reshaped the assessment to explicitly highlight C&CT, using the OECD rubric terminology that consisted of words related to C&CT (e.g. Table 1).

**Table 1:** Example of changes to the lesson plan and justification criterion for the common assessment task marking criteria over the duration of this study. Changes are shown with bolded words

Timing	Criterion description	Fail	Pass	Credit	Distinction	High distinction
Before study	A lesson plan to initiate a shift in learner thinking around the alternative conception(s) that incorporates activities (i.e. 2–3) that monitors learners’ understanding of the scientific concept and engage and extend the learner’s thinking	Poor structured lesson plan that does not satisfactorily address the alternative conception, lesson purpose and/or age appropriateness of the learner	Presents details of 2–3 learning activities relevant to the alternative conception are presented within the lesson plan	Presents details of 2–3 learning activities that adequately link alternative conceptions to the science concept in order to support learning. Clearly connects with findings from interview	Detailed lesson plan provided. Well-structured, relevant and detailed learning activities, informed by the interview data and literature, which address the alternative conceptions and encourage students thinking, reasoning and learning of the science concept are presented	The use of purposeful, detailed and logically structured learning activities, informed by the interview and literature, for specific contexts, such as addressing alternative conceptions are presented. These activities are used to explore, engage and extend the learners’ current understandings of the science concept
First year of study	Lesson plan and justification: Production and justification of a lesson plan to support a shift in learner’s thinking around the alternative conception/s and support student learning of the chosen science concept	Poorly structured or absent lesson plan that does not satisfactorily address the alternative conception, lesson purpose and/or age appropriateness of the learner Justification lacks sufficient support from relevant literature. **Limited or insufficient connection** to the interview	Lesson plan presents details of 2–3 learning activities relevant to the alternative conception which would benefit from further thinking and/or refinement to enhance effectiveness **Justification **shows evidence of support for the lesson plan which **connects **findings from the interview	Lesson plan presents 2–3 **pedagogically risky** activities that adequately link alternative conceptions to the science concept in order to support learning Supported justification for the lesson plan which clearly **connects** findings from the interview in light of the literature review	Detailed lesson plan presents well-structured, relevant, **pedagogically risky **and detailed learning activities which address the alternative conceptions to the science concept in order to support learning and encourage students thinking, reasoning and learning of the science concept. Well supported justification for the lesson plan which clearly **connects** findings from the interview and literature review and shows an understanding of the strengths of the plan	Purposeful, detailed, **pedagogically risky **and insightfully structured learning sequence which skillfully addresses the alternative conceptions and would support student thinking and learning about the science concept **Discerningly** supported justification for the lesson plan which insightfully **connects** findings from the interview and literature review and demonstrates a clear understanding of the strengths and limitations of the plan
Second year of study	Lesson plan: Production of a lesson plan to support a shift in learner thinking around the alternative conception/s and support student learning of the chosen science concept	Poorly structured or absent lesson plan that does not satisfactorily address the alternative conception. The lesson’s purpose is unclear and/or not age appropriate for the chosen level	Lesson plan presents details of planning, which is predominantly teacher centric. Activities are relevant to the alternative conception/s but would benefit from further **creative and critical thinking** and/or refinement to enhance effectiveness	Lesson plan presents pedagogical planning, which is somewhat teacher centric and would benefit from greater** creative and critical thinking** about how to make the tasks more student focused. Learning activities adequately address the alternative conception/s and may be useful for supporting student learning of the science concept	Detailed lesson plan presents well-structured, relevant, pedagogically planning, with some **originality**. Learning activities address the alternative conception/s and would support student learning of the science concept	Purposeful, detailed, and **original** pedagogical planning. Insightfully structured learning sequence which skillfully addresses the alternative conception/s and would support student thinking and learning about the science concept
	Lesson plan justification: Justification of the lesson plan	Justification lacks sufficient support from relevant literature. **Limited or insufficient connection** to the interview. Limited thinking about the potential effectiveness of the lesson plan	**Justification** shows evidence of support for the lesson plan which **connects** findings from the interview. Justification demonstrates some **creative and critical thinking **about the features of the plan	Supported **justification** for the lesson plan which clearly **connects** findings from the interview in light of the literature review. Justification demonstrates proficient **creative and critical thinking** about the features of the plan	Well supported justification for the lesson plan which **clearly connects **findings from the interview and literature review. Justification demonstrates an understanding of the strengths and/or limitations of the plan	**Discerningly **supported justification for the lesson plan which **insightfully connects** findings from the interview and literature review. Justification demonstrates a clear understanding of the strengths and limitations of the plan

Similarly, when we reviewed our science seminars, we found that C&CT was embedded in these seminars but not to the extent we thought:

One of the things that became obvious in the [OECD] workshops is that we are doing it [teaching for C&CT]. We are doing it in our seminars, throughout them, but we are not telling our students. (Rebecca, Meeting, Dec 2019).

Once teaching, we realised that we missed opportunities for explicitly teaching and assessing C&CT explicitly, and this recognition created unease. For instance, in terms of the common assessment task, we soon realised that the initial changes were not enough for PSTs to recognise and develop and understand that creative thinking was an essential part of the task. While the descriptors in the rubrics drew from the OECD language around creativity, we did not explicitly mention the term creativity. We were frustrated that we had missed and consequently downplayed such a central point, as we unintentionally undermined the role of creativity as integral to science teaching. We attended to this missed opportunity in the second year (see Table 1).

**Fig. 1:**
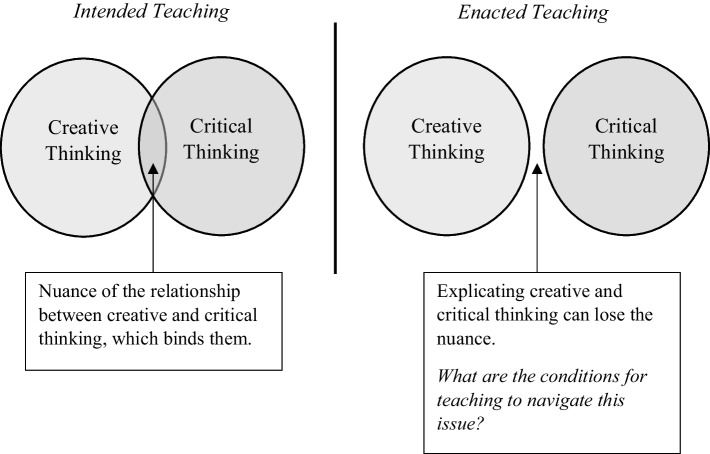
Intended and enacted teaching: what we intended to teach pre-service teachers about creative and critical thinking. However, explicating them for teaching assessment means that the nuance was sometimes lost, an issue we must navigate in our teaching (Meeting October, 2020)

Additionally, we became more sensitised to noticing when, how and why we encourage C&CT development in our science seminars as we challenged PSTs to reimagine their own understandings of science and science education. For example, we asked PSTs to identify what they saw as the purpose of science compared with science education, along with other prompts such as considering ways that science teachers may foster C&CT in their students when teaching different content. However, explicitly embedding C&CT into our teaching was not straightforward, particularly as we realised that explicating our thinking about C&CT with our secondary science PSTs was not always helpful. Clarification and articulation of C&CT into our teaching practice created tensions, where we felt that explication led to oversimplification of C&CT. For example, our common assessment task consisted of sequenced components, requiring PSTs to develop C&CT and incorporate their thinking in their task. As a teaching team, we regularly discussed ways to support PSTs’ learning without downplaying aspects of C&CT, as well as emphasising to PSTs the ways C&CT was needed to complete their final product. In one of our meetings (Oct, 2020), we drew the following item (Fig. 1) in response to these tensions we felt.

The left-hand side acknowledged the nuance and interplay (overlap) between creative and critical thinking, which is the learning we wanted our secondary science PSTs to develop. To support PSTs learning, we explicated our thinking to make this clearer to PSTs, and in doing so, we felt we separated C&CT apart. The nuance and interplay that binds C&CT was sometimes lost. Jared summarised:

We were continually playing jump rope between critical and creative thinking, as working together and as separate entities, as seen in the right-hand side of this diagram. But when we were explicit with either C or CT with the PSTs, we oversimplified the ways they work together. (Jared, Meeting, Oct 2020).

Drawing on our “jump rope” analogy, we noticed the “rope” became entangled when teaching C&CT. We attempted to untangle it by being explicit about “process and product” as well as “creativity *and* critical thinking”. We wondered if the interplay between C&CT was sometimes missed as attempts to inspire creativity in our seminars felt “forced”, and downplayed the complex, diverse and nuanced nature of these components. We came to realise that it is equally important to discuss the components separately, to consider each in turn to understand their individuality, and the interplay between them. We realised the need to draw attention to C&CT inherent in process and product, and what that means for science.

We continued to experience tensions about how to assess creativity and not lose the integrity of the creative process and its value in the final product. Creativity as a product does not equate to freedom and limitless possibilities; there are professional boundaries that must be considered, which Rebecca summarised as: “blue sky thinking still needs a net” (Rebecca, Meeting, Oct 2020). Another tension around assessment practices included concerns about stifling creativity. If we put too many parameters around the process/product and defined success around creativity in the marking rubric, we wondered what impact that might have on the ways students completed the task.

Consequently, we began seeking opportunities to explicitly identify and incorporate C&CT in our science seminars. We participated in a cyclical process of planning, doing and reflecting as we reviewed our planning and assessment. Scrutiny of our practice, particularly when forced to shift to online teaching, sensitised us to what we were already doing in relation to C&CT. Our group discussions were pivotal for noticing opportunities and unpacking how we could make C&CT more explicit for ourselves and our students, and importantly what it meant for teaching science to their future students. We continue to question if C&CT can (or should) be assessed in the same criterion. Is there always a clear distinction between C&CT? They often work together during processes, and are both necessary to form the final product, making C&CT difficult to delineate in an assessment. The tangled nature of C&CT needs to be addressed by TEs and communicated to PSTs to allow them to cultivate their own understandings of C&CT, including what it means for their future practice. Overtime, we came to recognise that while our initial explanations lacked nuance, persisting with explicitly embedding C&CT was worthwhile in the longer term.

### Theme Two: Developing a Shared Language and Understanding for Science Education

The OECD definitions for C&CT were helpful but were also too general. Discerning definitions forced us to deeply consider ways to make them fit with learning and teaching science. Furthermore, it became evident that while we used similar words (a shared language), we did not necessarily have the same purpose (a shared meaning). Despite a shared language, our understandings were often tacit and assumed, creating unintended tension within our teaching team. For example, adding the OECD term “pedagogically risky” to the assessment rubrics was problematic:

One of the terms was ‘pedagogically risky’… It caused some angst, and this was for a team member. So, I wonder how the students will respond to that*.* (Rebecca, Reflection, Dec 2019).

We have put in ‘pedagogically risky’ in order for it to be clear that we are talking about the language of creative thinking and that is what we are assessing. We have adapted the OECD language to add that language where we can, but we cannot put it all in. And, we are going to have to make sure we talk about that language and what it actually means? (Jennifer, Meeting, Dec 2019).

Shared understandings developed overtime, requiring deliberate consideration and deep reflective conversations. Consideration of differences in how we approached and talked about C&CT and how it looked in specific science methods was also important, especially since many of our PSTs studied two secondary science methods. For example, if we were going to use the term “pedagogically risky”, then we need to give examples of what this means in each science method, as well as being mindful of any confusion between methods. Expanding on the previous example:

I am mindful that psychology PSTs… have also studied ‘risky behaviours’ and risk-taking, mostly from a deficit default position, as harmful for mental health and wellbeing, or as risk-taking behaviours relative to an individual’s risk baseline. While ‘pedagogically’ can mean different things to different educators, ‘risky’ can mean different things… and for many [psychology PSTs], ‘risky behaviour’ equates to ‘dangerous behaviour’... While I want PSTs to take risks with their pedagogy, the term ‘pedagogically risky’ adds an extra layer of complexity*.* (Karen, Reflection, Dec 2019).

We identified needing to be on the same page, with a consistent approach, language and understanding, considering we had a shared seminar, shared assessment task, yet individual workshops for each discipline. The tensions we experienced led to clarification of appropriate forms of creativity when planning for learning and teaching science and an awareness for ourselves and PSTs about what creativity might look like in practice, both for teaching science and learning about science. For example, highlighting the creativity involved in finding ways to facilitate learning of science, beyond traditional transmissive methods, tapping into what students already know and understand. Teaching students to understand the role of creativity as part of the scientific endeavour, for instance, we asked PSTs to consider the role of creativity in science to help find a suitable vaccine for coronavirus, rather than simply following *THE* scientific method to get the answer. We became mindful of the contextual appropriateness of creative products, such as lesson plans and pedagogical approaches. Creativity is desirable for encouraging student engagement and learning, but as teachers, we need to exercise professional judgement and ensure creative outcomes are ethical, effective and contextually appropriate. Through these experiences, we reshaped the common assessment task in 2021 (e.g. Table 1).

### Theme Three: Illuminating the Conditions for Teaching Creative and Critical Thinking to Future Science Teachers

The final theme represents our professional learning in terms of creating the conditions for teaching C&CT to science PSTs. As TEs, we had to consider our expectations of PSTs as future science teachers, recognising the need to build PSTs’ confidence to employ C&CT to try new things when teaching science. This required establishing trusting relationships. We noticed PSTs were more likely to have a go if “permission” was given through immediate feedback, uncertainty was modelled by the teaching team, and C&CT was rewarded in their assessment.

We gave the students permission in the way we discussed C&CT in the seminars. They were allowed to do it, and we urged them to be innovative and, later, make and justify their final decisions (Karen, Reflection, May 2020).

We considered the important role of giving considerable time for discussion, and not rushing PSTs, as they came up with ideas of their own, such as ways to recognise students’ prior understanding of a science concept before teaching it, and then to share their experiences with others in the seminar for further inspiration and perspectives in an open and thoughtful way. We had to be careful not to stifle ideas, but rather withhold judgement and let them share different ideas and perspectives. We often asked them to re-engage with their groups and prompted them to collective decide on an idea and justify their decision. Building on these small group activities and whole class activities became important to promote C&CT, particularly creative thinking processes. The difficulties in separating C&CT, and then promoting the ways they work together to create the final product, became less problematic. In other words, we began to overcome the jump rope analogy (discussed earlier) as we recognised the conditions for teaching C&CT overtime.

For most PSTs, this approach worked well. However, a small group felt frustrated that we would not share the “correct” answer, the “correct” learning activity, or a “perfect” example of the assessment, which they believed would affect their final grade. Building trusting relationships worked both ways. We also had to trust our PSTs to engage with the activities, especially when they were working online, and when they were absent and engaging asynchronously.

Building conditions to nurture C&CT was an ongoing effort in the online seminar space, especially when PSTs were isolated as individuals, yet interacting with peers in the online seminar space. We noticed having to prioritise what creativity meant in science education, so that critical thinking did not overpower the creative process. PSTs too easily focused on critical thinking, which ended conversations, often prematurely. We also recognised the need to build excitement about learning and teaching C&CT and draw attention to C&CT in action, wherever possible. This was an added consideration for our practice.

## Discussion

This study considered the ways our professional knowledge was challenged, stimulated and developed as we worked together to explicitly embed C&CT in ITE to support our science PSTs to develop C&CT for their future teaching of secondary science. Examining our practice sensitised us to when, where, and how we were and could explicitly teach and assess C&CT within the context of science education. Integrating C&CT in more explicit ways was not as straight-forward as we first imagined. Initially, we found we had underplayed the role of creativity, such as not explicitly naming it in the assessment task, in line with other studies (Lorencová et al., 2019) and therefore this study extends the issue to science ITE. We embarked on this project already valuing C&CT and quickly became more cognizant about opportunities for teaching C&CT. We identified tensions, which led to development of a shared language and understanding of explicitly teaching and assessing C&CT in science education. Simultaneously, we developed a deeper understanding of the conditions required to facilitate PSTs learning, and in turn, their future students of science. Our findings show that teaching explicitly for C&CT in science ITE required deliberate planning, teaching and reflection together over time to transform our teaching practice.

Over time, we explicitly embedded C&CT in our practice to encourage PSTs to reimagine science education in a number of ways: first, in terms of the ways they connect C&CT with science, such as the nature and development of scientific knowledge and the use of science in our society; second, connecting C&CT with science education, such as thinking more broadly about the role of student-centred pedagogies in school science to make science more engaging and relevant to their future students’ lives; and finally, recognising the ways we were encouraging their C&CT development within our teaching practice and assessment. In particular, creating the conditions to facilitate and promote PSTs’ development of C&CT for teaching science required us to facilitate a culture of trust, along with helping PSTs overcome the perceived risk of failure to build confidence and take risks around thinking and teaching in creative ways.

Unless TEs are versed in the complexities of C&CT, their teaching may be limited. The OECD rubrics offered an excellent starting point to explicitly outline aspects of C&CT for us and our PSTs, but like others (Egan et al., 2017), we found ambiguity around definitions of C&CT. We had to delineate the terminology further in our science ITE classes. In the desire to teach C&CT in explicit ways, distinguishing between *teaching creativity* and *teaching for creativity* (Jeffrey and Craft, 2004) is required. Furthermore, we found that teaching PSTs to *learn to teach creatively* and *learn to teach for creativity* became important distinctions. Equally important was teaching PSTs to distinguish between *teaching critical thinking* and *teaching for critical thinking*, to *learn to teach critically* and *learn to teach for critical thinking*. We thought deeply about the *processes* and *products* of C&CT, and how these are reflected in assessments. Finally, as indicated early, each distinction demands an understanding of the role of C&CT in *science* and *science education* contexts.

This study contributes to understanding of the multiple barriers that can limit the teaching of C&CT in science ITE. In higher education, contextual barriers limit the teaching of C&CT (Marquis et al., 2017). Being explicit about C&CT forced the separation of C&CT in unintended and oversimplified ways. Initially, we may have unintentionally impaired or undermined creativity and its role in working with critical thinking when working on the assessment. Like Lorencová et al., (2019), we trialled active and collaborative learner centric strategies to support PSTs’ development. We realised we needed to devote time, space and dialogue for PSTs to collaborate, and share ideas and perspectives to cultivate the conditions for teaching C&CT. We found explicitly communicating C&CT and our justifications for teaching and assessments to PSTs helped set the conditions for embedding C&CT in purposeful ways over time. Small group and class discussions were important ways for PSTs to cultivate their own understanding of C&CT for science education and develop strategies to teach and assess their own science students’ C&CT. Collaborating in these ways were necessary to build trust between and within TEs and PSTs and take risks when embedding creativity in their assessment. While there are multiple barriers to teaching C&CT in higher education (Marquis et al., 2017), C&CT capabilities are more likely to develop when taught explicitly to PSTs (Lorencová et al., 2019).

## Conclusion, Limitations and Implications

Our study suggests that explicitly teaching C&CT in science ITE is a valuable but complex endeavour. Being a small-scale study, it is limited due to size and lack of generalisability. However, the complexities of teaching C&CT were somewhat amplified by the pandemic, forcing us to carefully consider our teaching and PSTs’ learning in deeper ways. Additionally, the scope of this study is limited to TEs voice. As we continue our journey to embed C&CT in our teaching, further research is needed to investigate how PSTs grapple with learning about and learning to teach C&CT as science teachers. Despite these limitations, our study describes our science TE professional learning in rich and contextualised ways. It provides ITE science education contexts and offers valuable insights into the role of collaborative critical reflection between science TEs to explicitly embed C&CT experiences into ITE science education. Other TEs may find our struggles and conditions for explicitly teaching and assessing C&CT useful in their own contexts.

The cultivation of understandings of and for teaching and learning C&CT explicitly in science ITE was not automatic nor an individual pursuit. Working together on the common seminars and the common assessment task, while also teaching different science subjects, enriched our learning and enabled collective reflection, leading to deep shared understandings about the nuanced nature of teaching C&CT explicitly within a science education context. While our different starting points, expertise and experiences created tensions, negotiating these was necessary to ease out the ways we framed C&CT learning experiences in our teaching practice. Assessment of C&CT explicitly in science ITE needs to clearly value PSTs’ growth and development that we as TEs are modelling and promoting in our teaching practice. Setting the conditions to trust each other, build and sustain trust with our PSTs and be clear in our expectations, task descriptions, rubrics, and feedback.

## Footnotes

1 One author moved universities during the writing of this paper.

2 OECD C&CT “Comprehensive Domain General” Rubrics can be located at: https://www.oecd.org/education/fostering-students-creativity-and-critical-thinking-62212c37-en.htm

## References

[CR1] Berry A, Vanderlinde R, Smith K, Murray J, Lunenberg M (2021). Interlude: Teacher educators’ professional development in Australia: Context and challenges. Teacher educators and their professional development: Learning from the past, looking to the future.

[CR2] Bryman, A. (2016). Social research methods (5th ed.). Oxford University Press.

[CR3] Carpendale, J., Delaney, S., & Rochette, E. (2020). Modeling meaningful chemistry teacher education online: Reflections from chemistry preservice teacher educators in Australia. *Journal of Chemical Education, 97*(9), 2534–2543. 10.1021/acs.jchemed.0c00718

[CR4] Cooper, R., Carpendale, J., Mansfield, J., & Marangio, K. (2020). *How teachers can embrace the rapid shift to online learning and teaching.* Monash Education TeachSpace. Monash University. https://www.monash.edu/education/teachspace/articles/how-teachers-can-embrace-the-rapid-shift-to-online-learning-and-teaching

[CR5] Craft A, Hall E, Wilson A (2014). Changes in the landscape for creativity in education. Creativity in Primary Education.

[CR6] Davies, M., & Barnett, R. (2015). Introduction. In M. Davies & R. Barnett (Eds.), *The Palgrave handbook of critical thinking in higher education* (pp. 1–26). Palgrave Macmillan. 10.1057/9781137378057

[CR7] Dennett DC (2013). Intuition pumps and other tools for thinking.

[CR8] Egan A, Maguire R, Christophers L, Rooney B (2017). Developing creativity in higher education for 21st century learners: A protocol for a scoping review. International Journal of Educational Research.

[CR9] Ellerton, P., & Kelly, R. (2021). Creativity and critical thinking. In A. Berry, C. Buntting, D. Corrigan, R. Gunstone, & A. Jones (Eds.), *Education in the 21st Century: STEM, Creativity and Critical Thinking* (pp. 9–27). Springer International Publishing. 10.1007/978-3-030-85300-6_2

[CR10] Elliott R, Timulak L, Miles J, Gilbert P (2005). Descriptive and interpretive approaches to qualitative research. Handbook of research methods for clinical and health psychology.

[CR11] Guest G, Bunce A, Johnson L (2006). How many interviews are enough? An experiment with data saturation and variability. Field Methods.

[CR12] Hadzigeorgiou, Y. (2016). *Imaginative Science Education: The Central Role of Imagination in Science Education* (1st ed. 2016. ed.). Springer. 10.1007/978-3-319-29526-8

[CR13] Janssen EM, Mainhard T, Buisman RSM, Verkoeijen PPJL, Heijltjes AEG, van Peppen LM, van Gog T (2019). Training higher education teachers’ critical thinking and attitudes towards teaching it. Contemporary Educational Psychology.

[CR14] Jeffrey B, Craft A (2004). Teaching creatively and teaching for creativity: Distinctions and relationships. Educational Studies.

[CR15] Kind PM, Kind V (2007). Creativity in science education: Perspectives and challenges for developing school science. Studies in Science Education.

[CR16] Knight PT (2002). Small-scale Research. SAGE.

[CR17] Lorencová H, Jarošová E, Avgitidou S, Dimitriadou C (2019). Critical thinking practices in teacher education programmes: A systematic review. Studies in Higher Education.

[CR18] Mansfield, J., & Gunstone, R. (2021). When failure means success: accounts of the role of failure in the development of new knowledge in the STEM disciplines. In A. Berry, C. Buntting, D. Corrigan, R. Gunstone, & A. Jones (Eds.), *Education in the 21st Century: STEM, Creativity and Critical Thinking *(pp. 137–158). Springer. 10.1007/978-3-030-85300-6_9

[CR19] Marangio, K., Mansfield, J., Carpendale, J., & Cooper, R. (2021). *Making the most of our virtual classroom.* Monash Education TeachSpace. Monash University. https://www.monash.edu/education/teachspace/articles/making-the-most-of-our-virtual-classroom

[CR20] Marquis E, Radan K, Liu A (2017). A present absence: Undergraduate course outlines and the development of student creativity across disciplines. Teaching in Higher Education.

[CR21] Patston TJ, Cropley DH, Marrone RL, Kaufman JC (2018). Teacher implicit beliefs of creativity: Is there an arts bias?. Teaching and Teacher Education.

[CR22] Patston TJ, Kaufman JC, Cropley AJ, Marrone R (2021). What is creativity in education? A qualitative study of international curricula. Journal of Advanced Academics.

[CR23] Poulson, L., & Wallace, M. (2003). Designing and writing about research. In L. Poulson, & M. Wallace (Eds.), *Learning to read critically in teaching and learning.* (pp. 37–50). Thousand Oaks: SAGE Publications

[CR24] Sammel A (2014). Science as a human endeavour: Outlining scientific literacy and rethinking why we teach science. Creative Education.

[CR25] Skinner R, Foulds W, Cousins J (1994). The effect of intervention strategies on creative thinking skills of pre-service teachers. Research in Science Education.

[CR26] Vincent-Lancrin S, González-Sancho C, Bouckaert M, Luca FD, Fernández-Barrerra M, Jacotin G, Urgel J, Vidal Q (2019). Fostering students’ creativity and critical thinking: What it means in school. Centre for Educational Research and Innovation OECD.

[CR27] Walsh E, Anders K, Hancock S, Elvidge L (2013). Reclaiming creativity in the era of impact: Exploring ideas about creative research in science and engineering. Studies in Higher Education.

[CR28] Watson J (2018). Deferred creativity: Exploring the impact of an undergraduate learning experience on professional practice. Teaching and Teacher Education.

